# Supplementary material for: Connexin 30 Deficiency Ameliorates Disease Progression at the Early Phase in a Mouse Model of Amyotrophic Lateral Sclerosis by Suppressing Glial Inflammation
Source: Int J Mol Sci. 2022 Dec 16;23(24):16046. doi: 10.3390/ijms232416046 (PMC9782489; doi:10.3390/ijms232416046)
Supplement: Supplementary file 1 [file ijms-23-16046-s001.zip › ijms-1969786-supplementary.pdf]

# Supplementary Figure S1

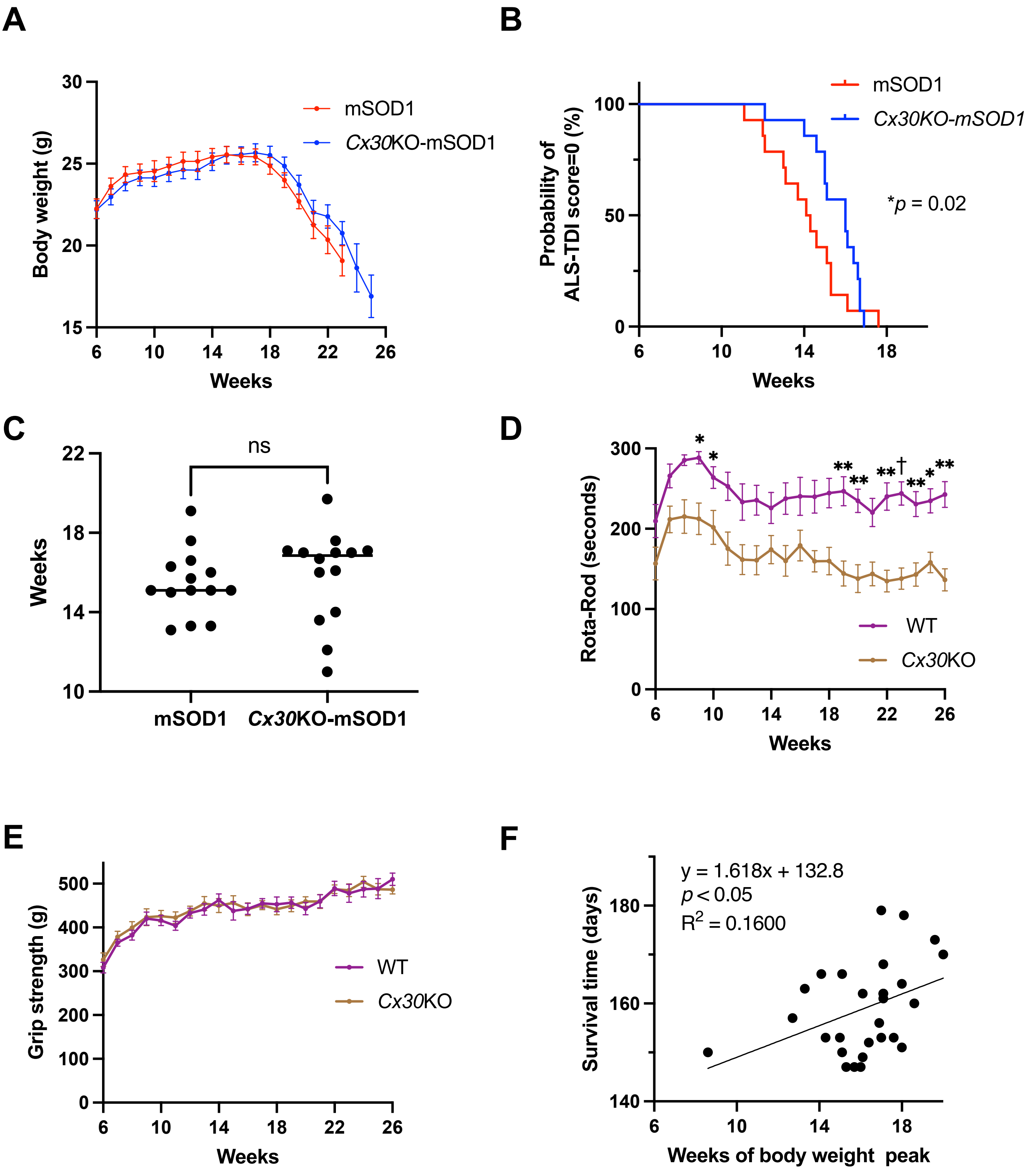


Supplementary Figure S1. Phenotypic features of Cx30KO-mSOD1, mSOD1, Cx30KO, and WT mice. Red and blues lines indicate mSOD1 and Cx30KO-mSOD1 mice, respectively. Purple and brown lines indicate WT and Cx30KO mice, respectively. (A) Body weights of mSOD1 and Cx30KO-mSOD1 mice. (B) The time curve to reach ALS-TDI score = 1. (C) Comparison of the 20% reduction from the peak in the rotarod test between mSOD1 and Cx30KO-mSOD1 mice. (D) The rotarod test in WT and Cx30KO mice. (E) Grip strength of WT and Cx30KO mice. (F) Relationship between survival time and timing of body weight peak. The Gehan-Breslow-Wilcoxon test was used to statistically compare the timing of ALS-TDI score = 1 ($p$ = 0.02). The Kolmogorov−Smirnov test assessed the reduction from the peak of the rotarod test. Multiple t-tests were used for statistical comparisons of the rotarod test data. Simple linear regression was used to analyze the relationship between survival time and body weight peak. ns = not significant, *$p < 0.05$, **$p < 0.01$, and †$p < 0.001$.

# Supplementary Figure S2

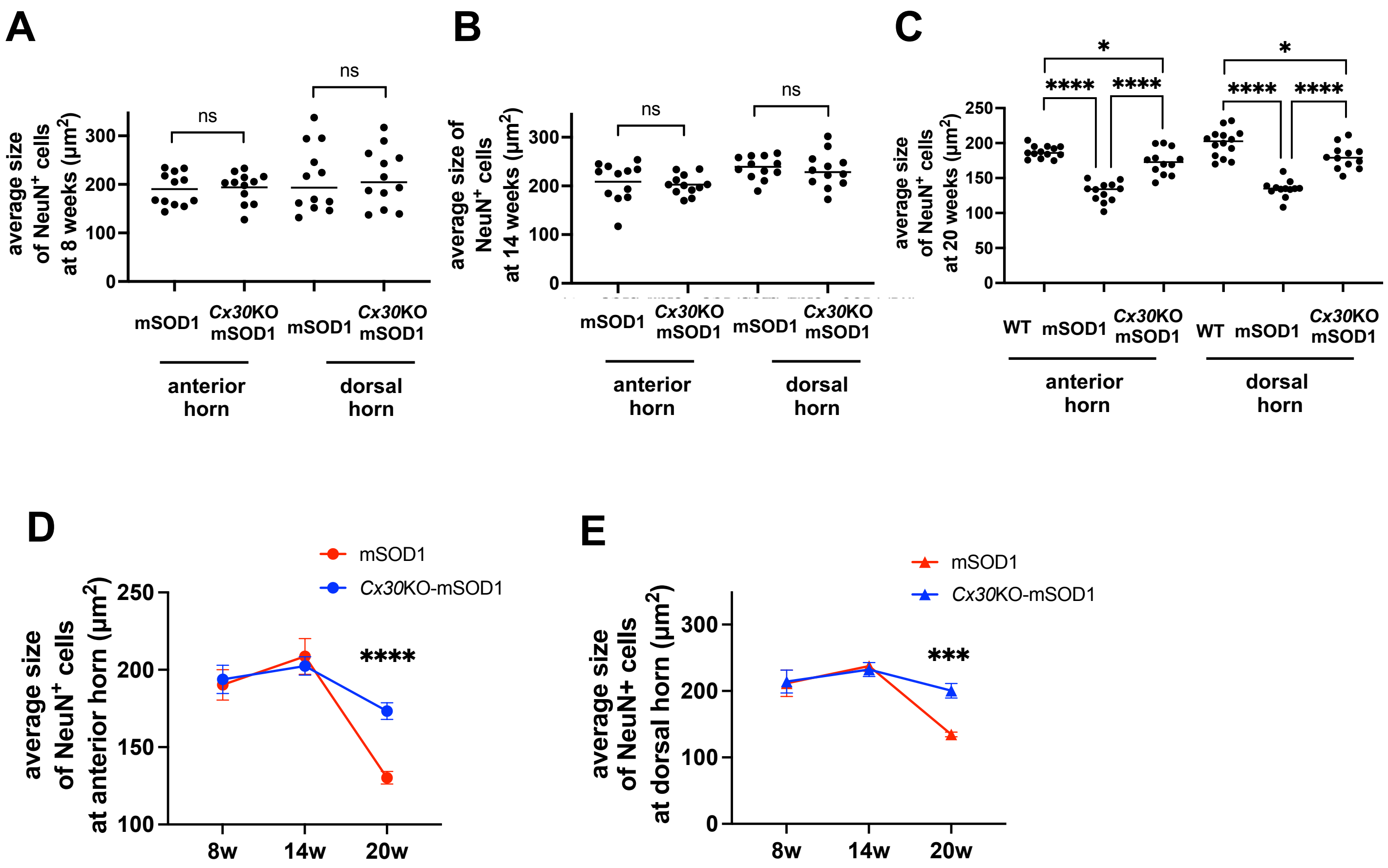


Supplementary Figure S2. (A, B, C) Quantification of the size of anterior horn cells and dorsal horn cells in WT, mSOD1, and Cx30KO-mSOD1 mice at 8, 14, and 20 weeks. n = 12 (4 slices from every 3 mice). (D, E) The time course of anterior and dorsal horn cell size in each group. Red and blue lines indicate mSOD1 and Cx30KO-mSOD1 mice, respectively. Statistical differences were assessed using an unpaired t-test with Welch's correction for the cell size at 8 and 14 weeks. Welch's ANOVA plus Dunnett's T3 multiple comparison tests were used for the cell size at 20 weeks. The time course of cell size was evaluated with two-way ANOVA plus Sidak's multiple comparison test. ns = not significant. $^*p < 0.05$, $^{***}p < 0.001$, and $^{****}p < 0.0001$.

# Supplementary Figure S3

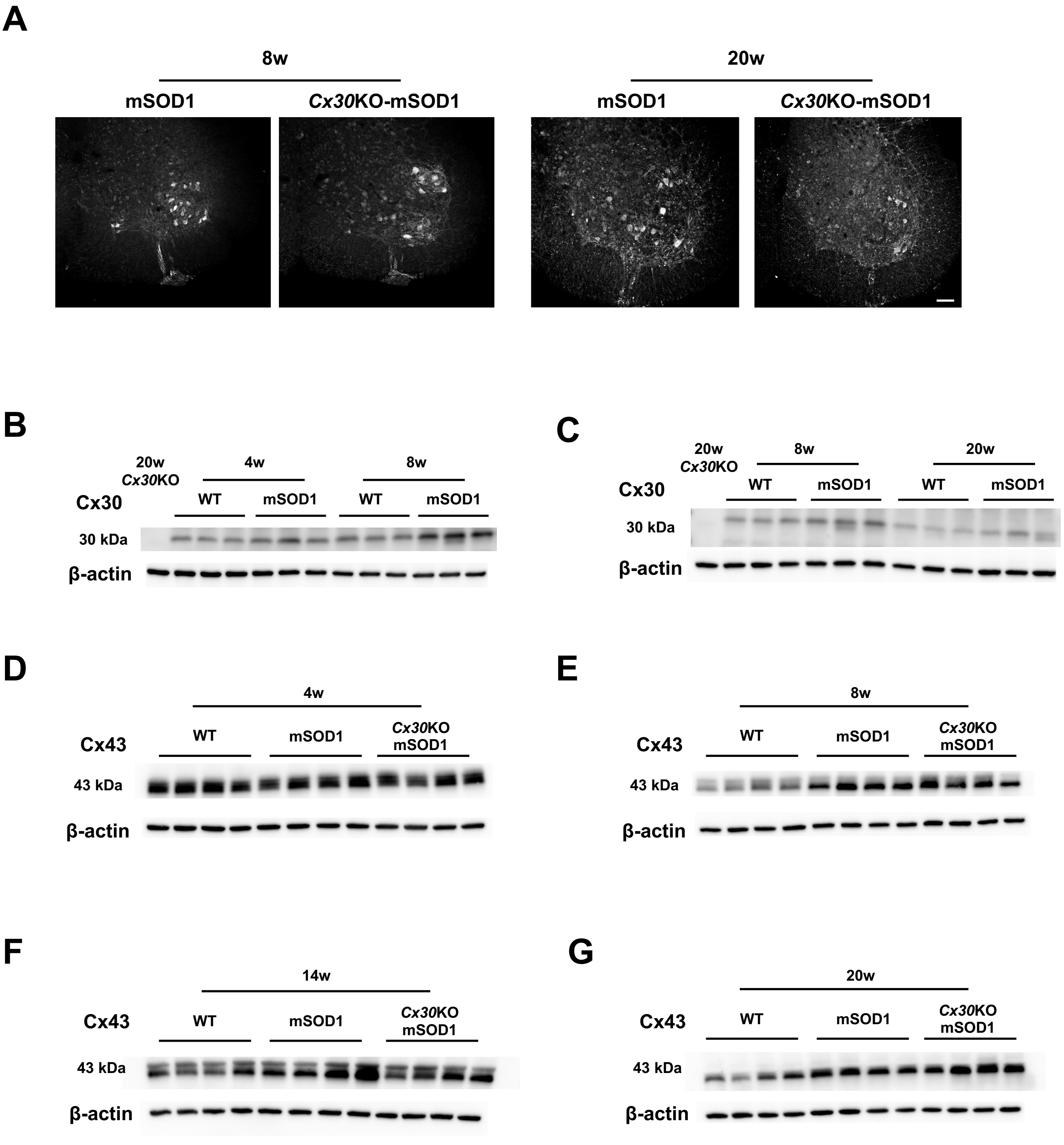


Supplementary Figure S3. (A) mSOD1 immunostaining in the lumbar spinal cord in mSOD1 and Cx30KO-mSOD1 mice at 8 and 20 weeks. (B, C) Representative western blot images of Cx30 between 4 and 8 weeks and 8 and 20 weeks, respectively. (D, E, F, G) Representative western blot images of Cx43 at 4, 8, 14, and 20 weeks, respectively.

# Supplementary Figure S4

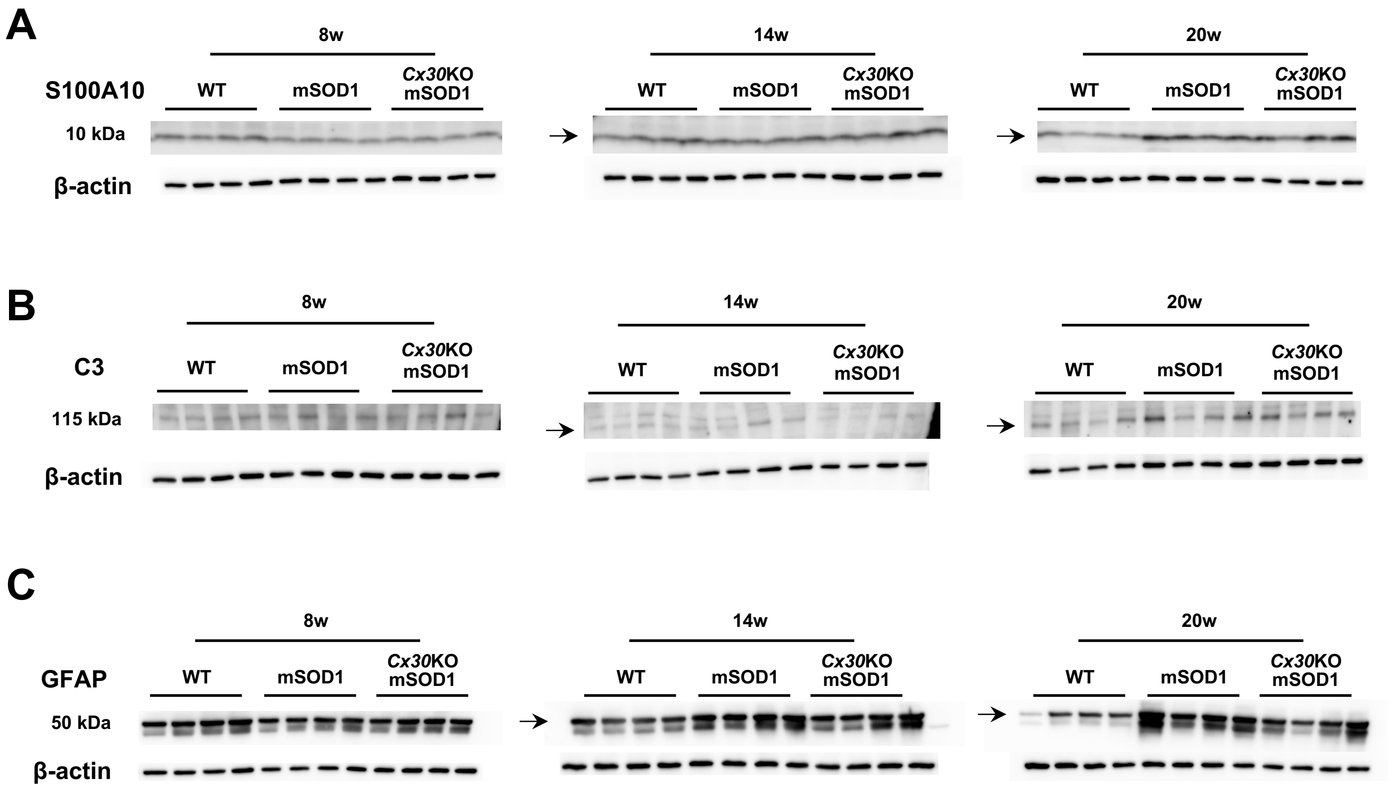


Supplementary Figure S4. (A, B, C) Representative western blot images of S100A10 (A), C3 (B), and GFAP (C) at 8, 14, and 20 weeks. Arrows show the target bands.

# Supplementary Figure S5

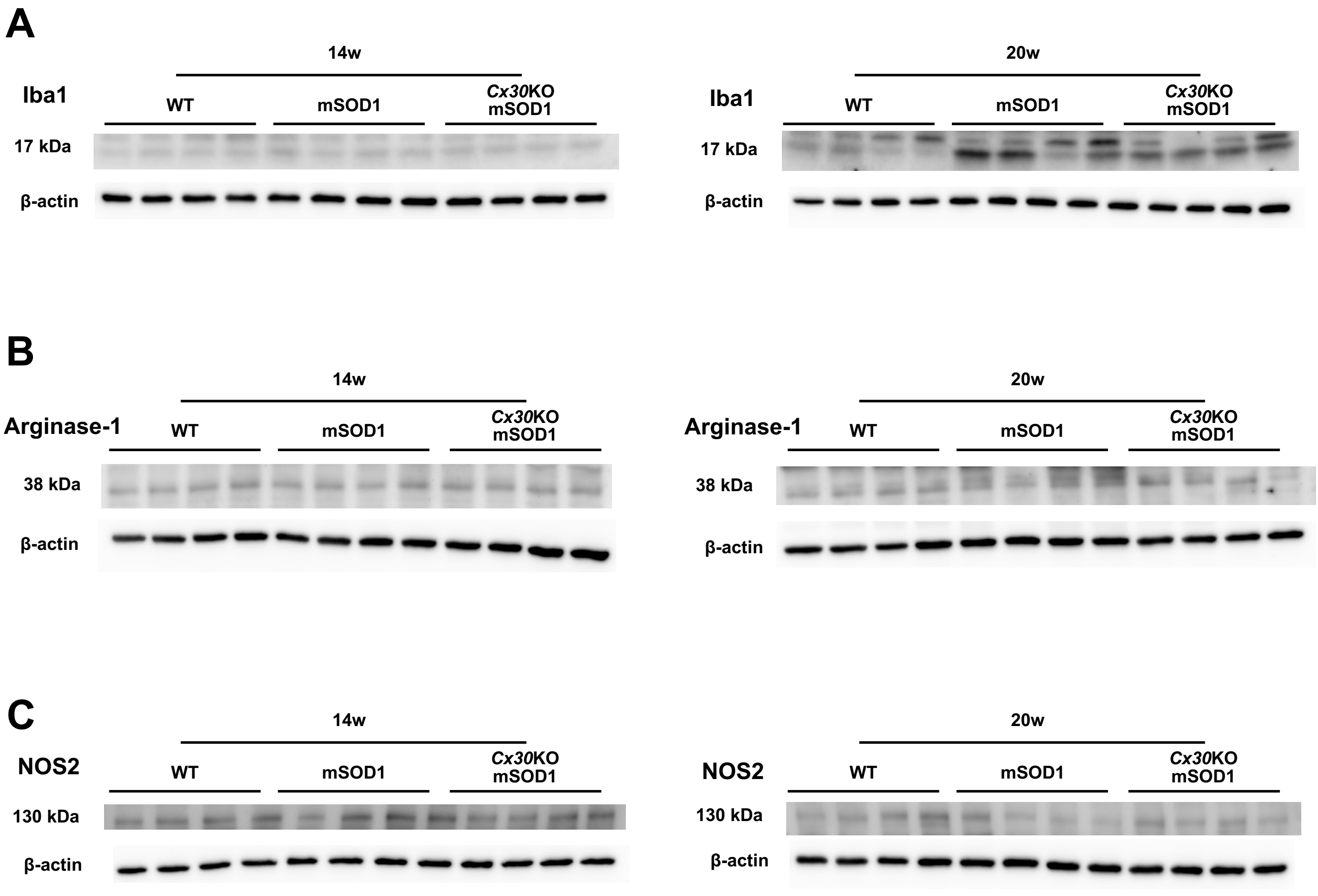


Supplementary Figure S5. (A, B, C) Representative western blot images of Iba1 (A), Arg1 (B), and NOS2 (C) at 14 and 20 weeks.

# Supplementary Table S1

Immunohistochemistry

| Antibody | Host | Clonality | Dilution | Supplier | Catalog |
|---|---|---|---|---|---|
| NeuN | mouse | monoclonal | 1:5000 | MILLIPORE | MAB377 |
| mSOD1 | rat | monoclonal | 1:500 | MILLIPORE | MABN834 |
| GFAP | mouse | monoclonal | 1:1000 | STEMCELL | 60048 |
| Cx30 | rabbit | polyclonal | 1:1000 | Invitrogen | 712200 |
| Cx43 | rabbit | polyclonal | 1:1000 | abcam | ab11370 |
| S100A10 | goat | polyclonal | 1:100 | R&D systems | AF2377 |
| C3 | rat | monoclonal | 1:250 | Hycult Biotech | HM1045 |
| Iba1 | rabbit | polyclonal | 1:500 | Wako | 019-19741 |
| Arginase-1 | mouse | monoclonal | 1:250 | SANTA CLUZ | sc-271430 |
| NOS2 | rabbit | polyclonal | 1:200 | Bioss | bs-2072R |

Western blotting

| Antibody | Host | Clonality | Dilution | Supplier | catalog |
|---|---|---|---|---|---|
| Cx30 | mouse | monoclonal | 1:500 | SANTA CLUZ | sc-514847 |
| Cx43 | rabbit | polyclonal | 1:20000 | abcam | ab11370 |
| GFAP | mouse | monoclonal | 1:10000 | STEMCELL | 60048 |
| S100A10 | goat | polyclonal | 1:1000 | R&D systems | AF2377 |
| C3 | rabbit | polyclonal | 1:2000 | abcam | ab200999 |
| Iba1 | rabbit | polyclonal | 1:500 | Wako | 016-20001 |
| Arginase-1 | mouse | monoclonal | 1:100 | SANTA CLUZ | sc-271430 |
| NOS2 | rabbit | polyclonal | 1:667 | Bioss | bs-2072R |
| β-actin | mouse | monoclonal | 1:20000 | Sigma-aldrich | A5441 |

NeuN = neuronal nuclei; mSOD1 = mutant superoxide dismutase-1; GFAP = glial fibrillary acidic protein; Cx = connexin; Iba1 = ionized calcium-binding adapter molecule 1; NOS2 = nitric oxide synthase 2
